# Attitudes of Psychiatric Hospital Staff Toward Restraint and Influencing Factors

**DOI:** 10.1192/j.eurpsy.2025.1577

**Published:** 2025-08-26

**Authors:** I. H. Cho, I. Sohn

**Affiliations:** 1 Psychiatry, WIM Psychiatric Clinic, Seongnam; 2 Psychiatry, Keyo Hospital, Uiwang, Korea, Republic Of

## Abstract

**Introduction:**

There has been an increasing socio-medical discourse on the humanitarian approach to the use of restraint in psychiatric inpatient units.

**Objectives:**

It is necessary to investigate the attitudes of psychiatric hospital staff toward the use of restraint in psychiatric inpatient units and to identify the factors influencing these attitudes.

**Methods:**

This study examined the attitudes of psychiatric hospital staff toward the use of restraint in situations involving physical violence toward other patients, verbal violence toward other patients, physical violence toward staff, verbal violence toward staff, and disruption of the treatment environment. The study also investigated factors related to the considerations and perceived burdens (both legal and medical) associated with the use of restraint, comparing these findings with data from a survey conducted 10 years ago.Attitudes toward restraint were not significantly associated with gender, age, or years of service. However, staffs who exhibited less prejudice toward mental illness-related crime were less likely to find restraint necessary. Compared to 10 years ago, there was little change in the need for restraint in cases of physical violence (both toward patients and staff ), but the need for restraint in response to verbal violence (toward both patients and staff ) had decreased. The perceived burden, both legal and medical, associated with the use of restraint had increased.

**Results:**

Attitudes toward restraint were not significantly associated with gender, age, or years of service. However, staff who exhibited less prejudice toward mental illness-related crime were less likely to find restraint necessary. Compared to 10 years ago, there was little change in the need for restraint in cases of physical violence (both toward patients and staff ), but the need for restraint in response to verbal violence (toward both patients and staff ) had decreased. The perceived burden, both legal and medical, associated with the use of restraint had increased.

**Conclusions:**

Psychiatric hospital staffs with less prejudice toward mental illness-related crime were less likely to perceive a need for the use of restraint. Compared to 10 years ago, the necessity of restraint in cases of verbal violence has decreased, which may be attributed to ongoing human rights education and increased legal and medical concerns. These findings provide important insights for future policy development aimed at promoting humanitarian approaches, such as non-restraint treatments.

**Disclosure of Interest:**

None Declared

